# Re: Re: Laparoscopic ovarian transposition and ovariopexy for fertility preservation in patients treated with pelvic radiotherapy with or without chemotherapy

**Published:** 2020-05-07

**Authors:** Lale Turkgeldi, Engin Turkgeldi, Ali Alchami, Alison Nichol, Ertan Saridogan

**Affiliations:** University College London Hospital and UCL Institute for Women’s Health. London, UK.

Dear Editor,

We would like to thank Drs. Torres-de la Roche and De Wilde for their kind comments on our recently published paper “Laparoscopic ovarian transposition and ovariopexy for fertility preservation in patients treated with pelvic radiotherapy with or without chemotherapy” (Turkgeldi et al., 2019) and for bringing their contribution to the literature of perhaps one of the earliest publications concerning laparoscopic oophoropexy prior to radiotherapy in patients with Hodgkins lymphoma to our attention (De Wilde and Hesseling, 1995).

The promising results of this pioneering prospective comparative study have undoubtedly encouraged many gynaecologists to take steps towards performing minimally invasive surgery for the preservation of hormonal and reproductive function in patients with various pelvic malignancies requiring pelvic radiotherapy.

As mentioned by Dr. Torres-de la Roche and Dr. De Wilde, other methods of fertility preservation including oocyte or ovarian tissue cryopreservation and gonadotropin agonist therapy are being utilized as additional measures to preserve ovarian function in cancer patients, especially for those who will receive chemotherapy. The implementation of GnRH antagonists along with GnRH agonists to decrease the initial FSH flare-up effect prior to chemotherapy may have beneficial effects, however further studies are necessary to investigate the true impact of medical treatment in preserving ovarian function (Von Wolff et al., 2010).

Laparoscopic ovarian transposition is a safe and effective surgical procedure for optimizing fertility preservation in patients undergoing pelvic radiotherapy when implemented alone or in combination with other surgical, medical or assisted reproductive treatment modalities (Moawad et al., 2016). It is important that premenopausal cancer patients are well informed about their fertility options since life expectancy after cancer treatment has been increasing progressively with recent advancements in medicine (Miller et al., 2016).

Lale Turkgeldi, Engin Turkgeldi, Ali Alchami, Alison Nichol, Ertan Saridogan, University College London Hospital and UCL Institute for Women’s Health. London, UK. E-Mail: ertan.saridogan@nhs.net
